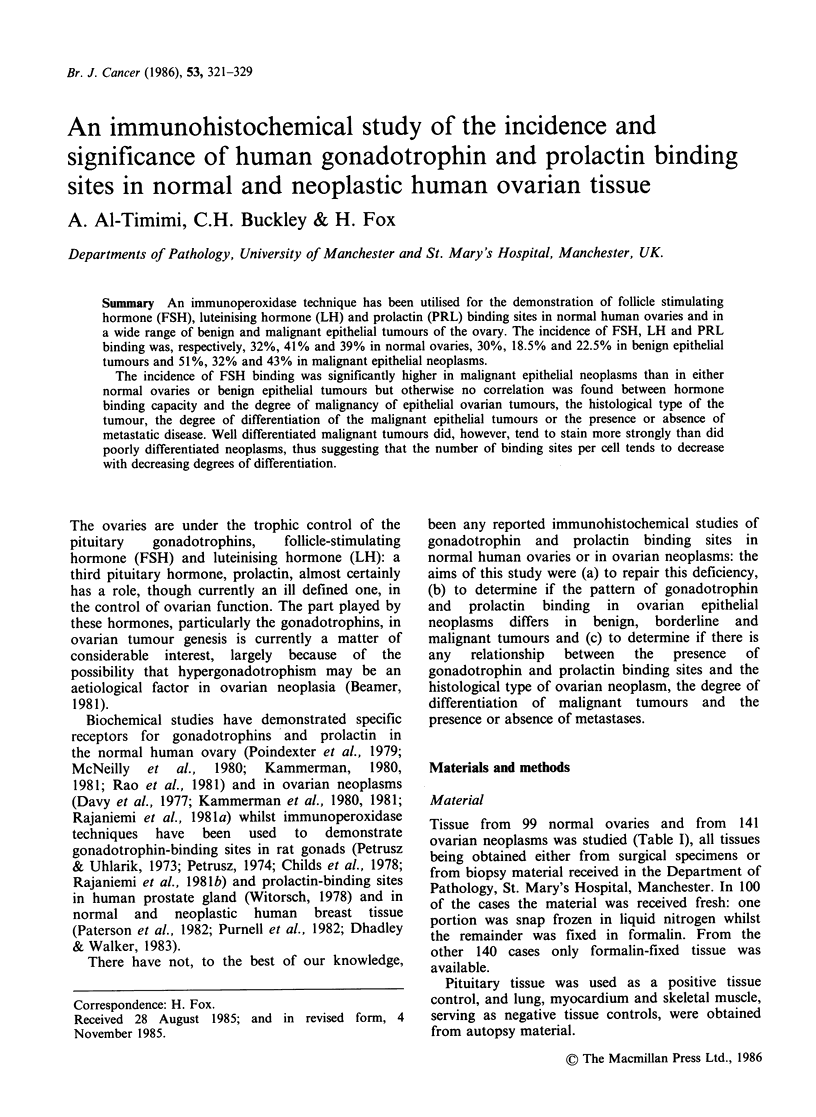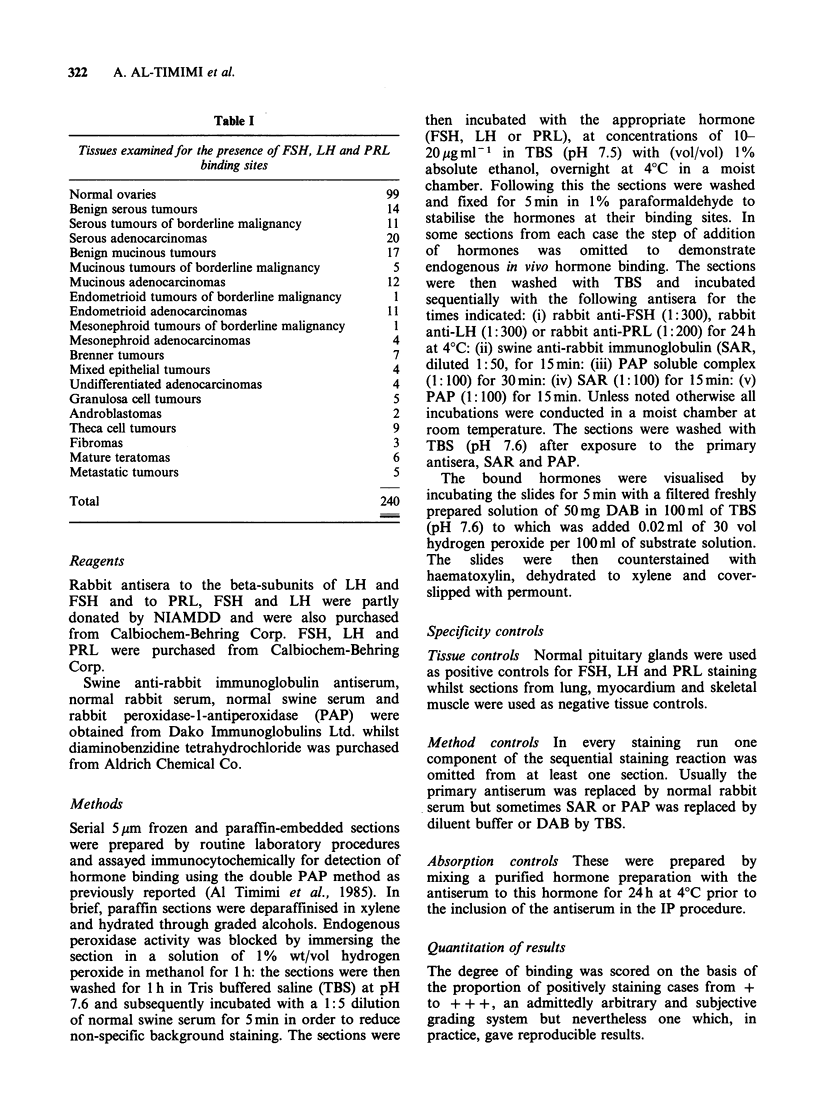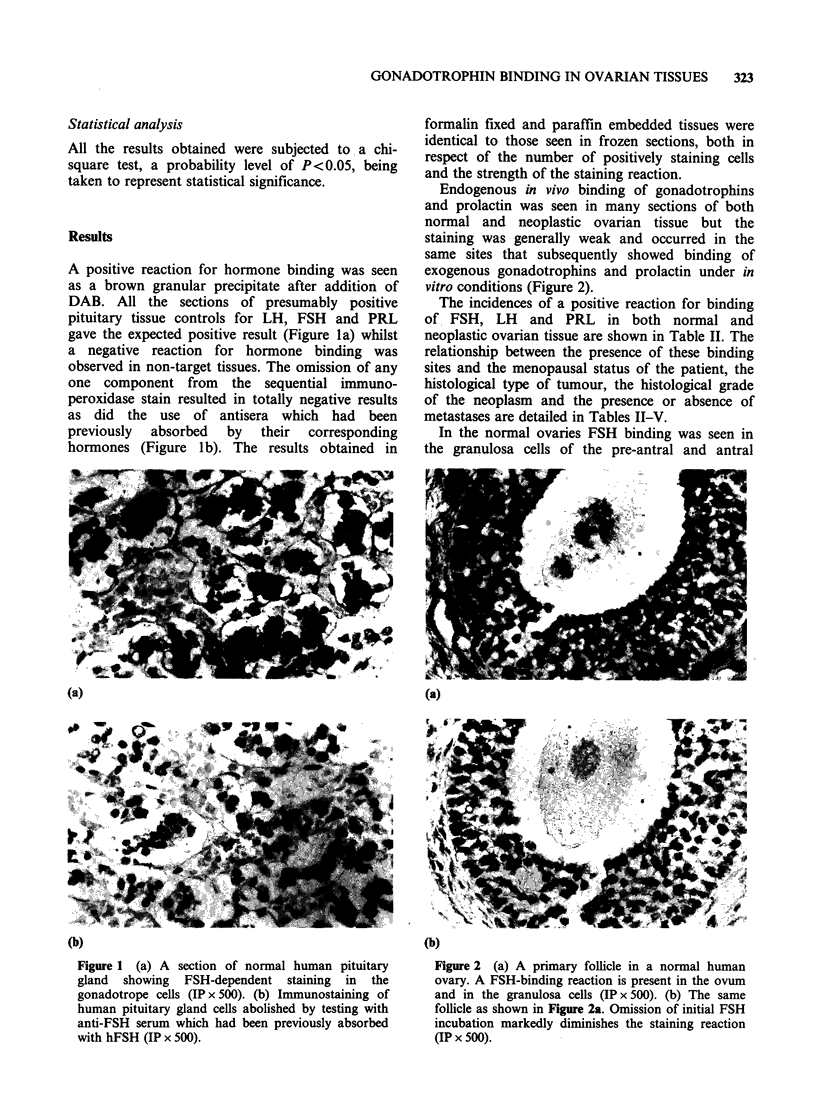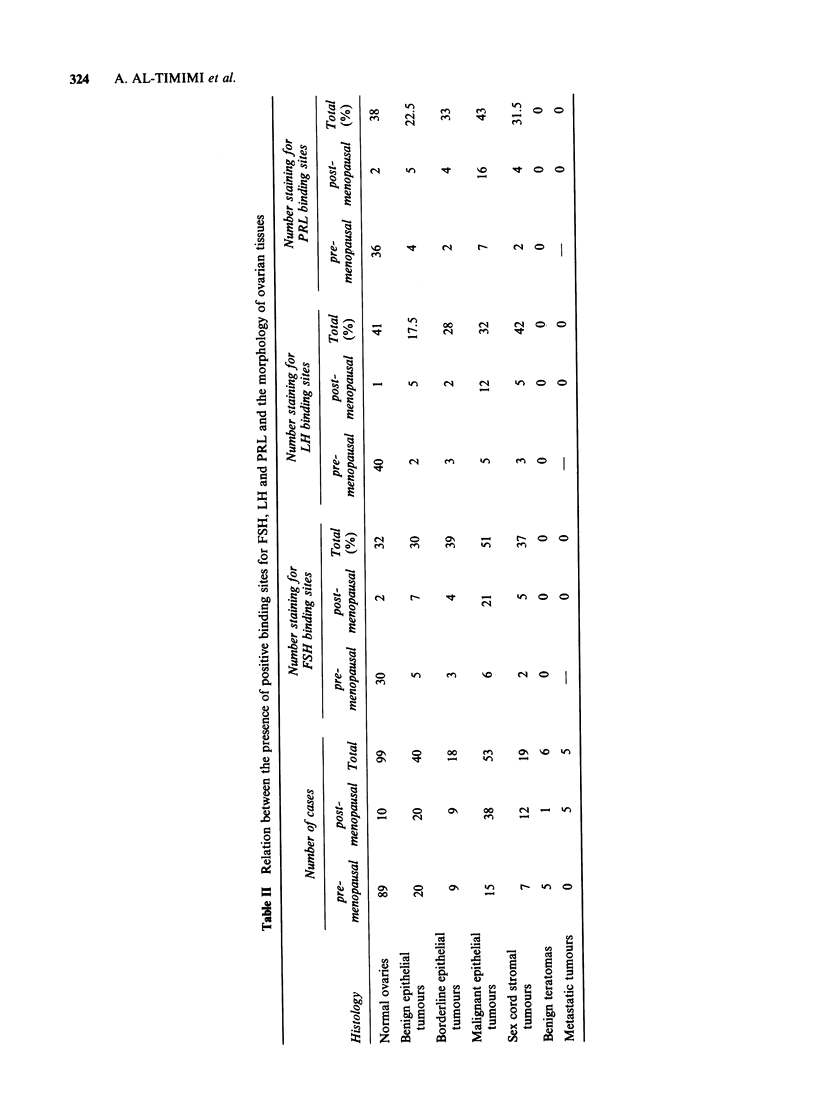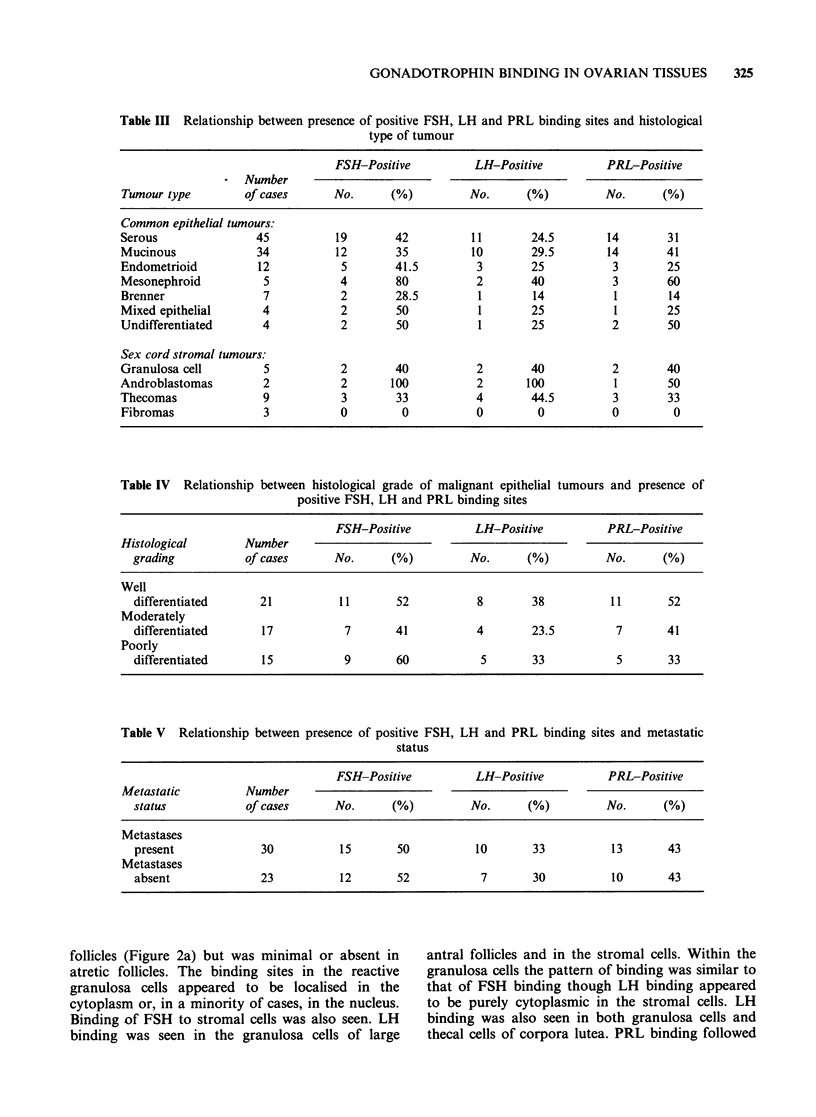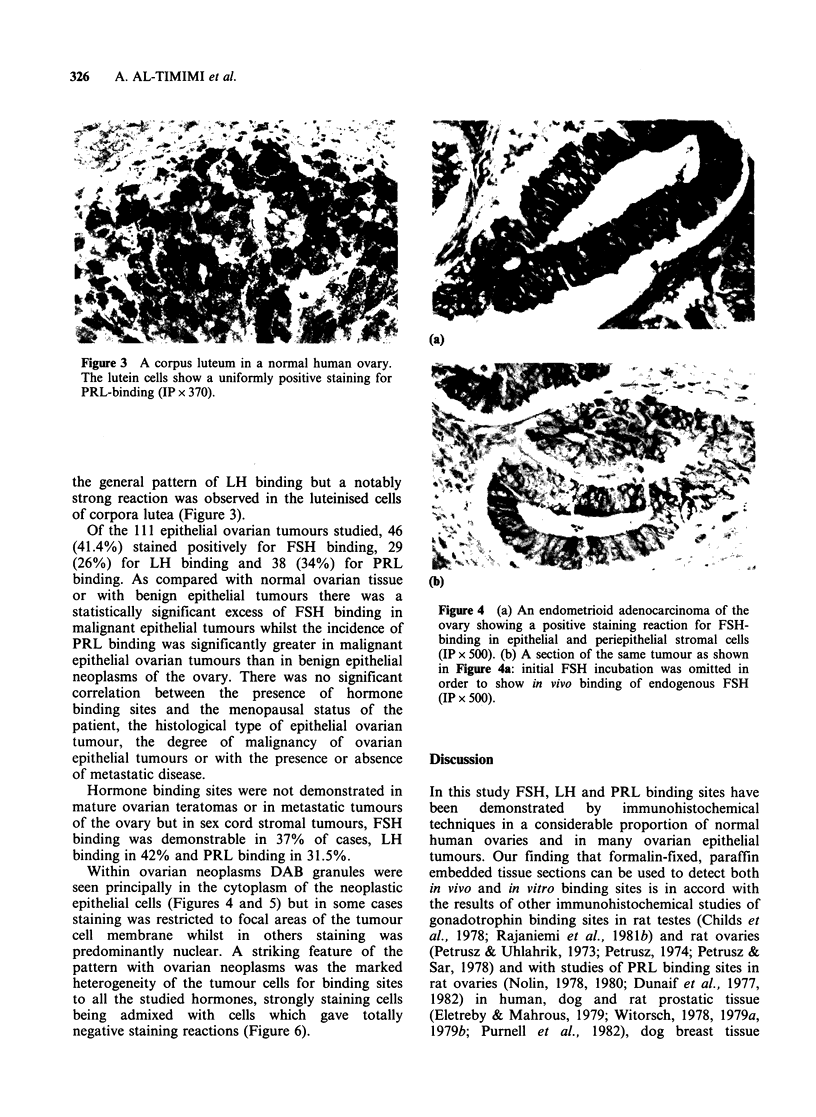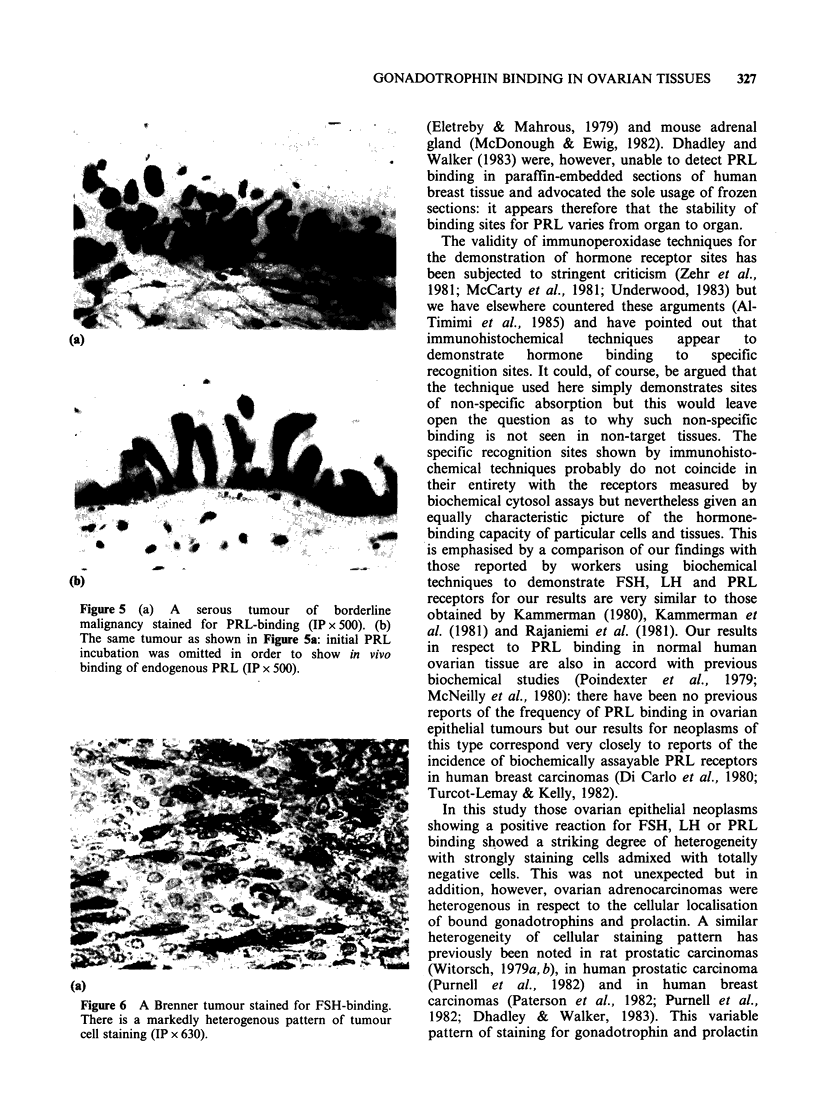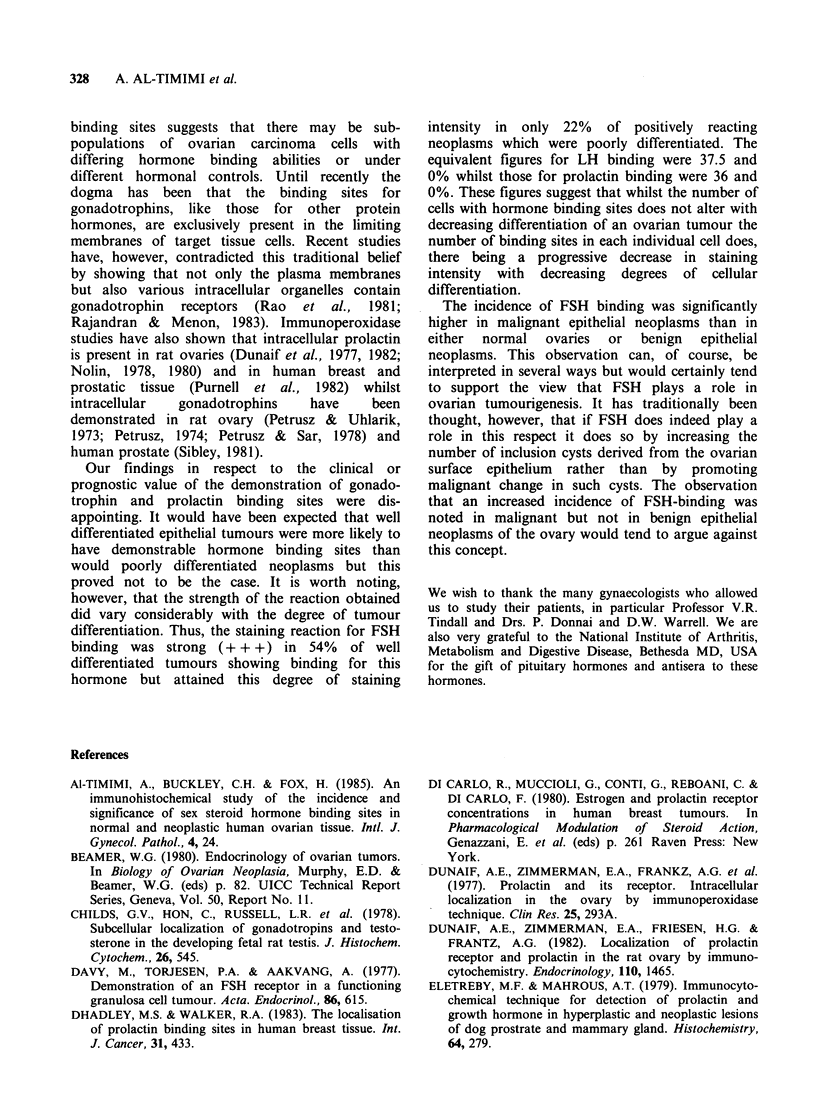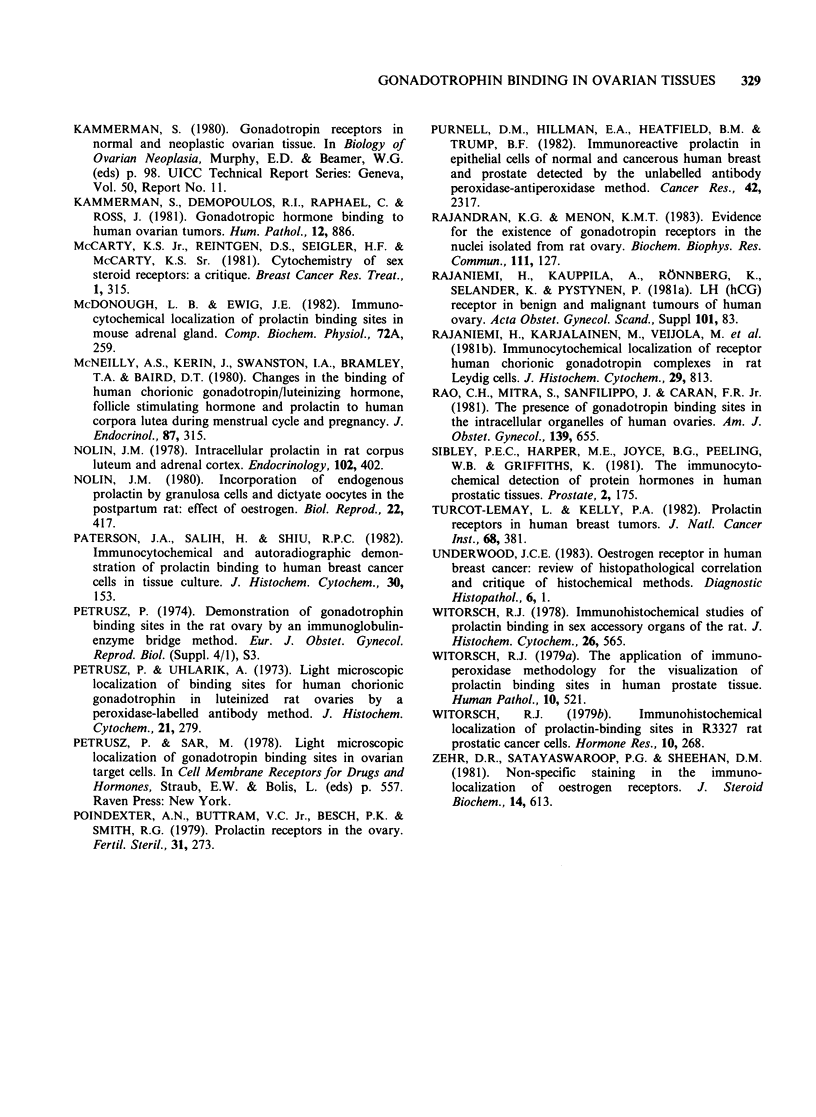# An immunohistochemical study of the incidence and significance of human gonadotrophin and prolactin binding sites in normal and neoplastic human ovarian tissue.

**DOI:** 10.1038/bjc.1986.55

**Published:** 1986-03

**Authors:** A. Al-Timimi, C. H. Buckley, H. Fox

## Abstract

**Images:**


*Br. J. Cancer* (1986), **53**, 321–329

### An immunohistochemical study of the incidence and significance of human gonadotrophin and prolactin binding sites in normal and neoplastic human ovarian tissue

A. Al-Timimi, C.H. Buckley & H. Fox

*Departments of Pathology, University of Manchester and St. Mary's Hospital, Manchester, UK.*

**Summary** An immunoperoxidase technique has been utilised for the demonstration of follicle stimulating hormone (FSH), luteinising hormone (LH) and prolactin (PRL) binding sites in normal human ovaries and in a wide range of benign and malignant epithelial tumours of the ovary. The incidence of FSH, LH and PRL binding was, respectively, 32%, 41% and 39% in normal ovaries, 30%, 18.5% and 22.5% in benign epithelial tumours and 51%, 32% and 43% in malignant epithelial neoplasms.

The incidence of FSH binding was significantly higher in malignant epithelial neoplasms than in either normal ovaries or benign epithelial tumours but otherwise no correlation was found between hormone binding capacity and the degree of malignancy of epithelial ovarian tumours, the histological type of the tumour, the degree of differentiation of the malignant epithelial tumours or the presence or absence of metastatic disease. Well differentiated malignant tumours did, however, tend to stain more strongly than did poorly differentiated neoplasms, thus suggesting that the number of binding sites per cell tends to decrease with decreasing degrees of differentiation.

The ovaries are under the trophic control of the pituitary gonadotrophins, follicle-stimulating hormone (FSH) and luteinising hormone (LH): a third pituitary hormone, prolactin, almost certainly has a role, though currently an ill defined one, in the control of ovarian function. The part played by these hormones, particularly the gonadotrophins, in ovarian tumour genesis is currently a matter of considerable interest, largely because of the possibility that hypergonadotrophism may be an aetiological factor in ovarian neoplasia (Beamer, 1981).

Biochemical studies have demonstrated specific receptors for gonadotrophins and prolactin in the normal human ovary (Poindexter *et al.*, 1979; McNeilly *et al.*, 1980; Kammerman, 1980, 1981; Rao *et al.*, 1981) and in ovarian neoplasms (Davy *et al.*, 1977; Kammerman *et al.*, 1980, 1981; Rajaniemi *et al.*, 1981*a*) whilst immunoperoxidase techniques have been used to demonstrate gonadotrophin-binding sites in rat gonads (Petrusz & Uhlarik, 1973; Petrusz, 1974; Childs *et al.*, 1978; Rajaniemi *et al.*, 1981*b*) and prolactin-binding sites in human prostate gland (Witorsch, 1978) and in normal and neoplastic human breast tissue (Paterson *et al.*, 1982; Purnell *et al.*, 1982; Dhadley & Walker, 1983).

There have not, to the best of our knowledge, been any reported immunohistochemical studies of gonadotrophin and prolactin binding sites in normal human ovaries or in ovarian neoplasms: the aims of this study were (a) to repair this deficiency, (b) to determine if the pattern of gonadotrophin and prolactin binding in ovarian epithelial neoplasms differs in benign, borderline and malignant tumours and (c) to determine if there is any relationship between the presence of gonadotrophin and prolactin binding sites and the histological type of ovarian neoplasm, the degree of differentiation of malignant tumours and the presence or absence of metastases.

Correspondence: H. Fox.
Received 28 August 1985; and in revised form, 4 November 1985.

### Materials and methods

### *Material*

Tissue from 99 normal ovaries and from 141 ovarian neoplasms was studied (Table I), all tissues being obtained either from surgical specimens or from biopsy material received in the Department of Pathology, St. Mary's Hospital, Manchester. In 100 of the cases the material was received fresh: one portion was snap frozen in liquid nitrogen whilst the remainder was fixed in formalin. From the other 140 cases only formalin-fixed tissue was available.

Pituitary tissue was used as a positive tissue control, and lung, myocardium and skeletal muscle, serving as negative tissue controls, were obtained from autopsy material.

© The Macmillan Press Ltd., 1986

**Table I**

*Tissues examined for the presence of FSH, LH and PRL binding sites*

| | |
|---|---|
| Normal ovaries | 99 |
| Benign serous tumours | 14 |
| Serous tumours of borderline malignancy | 11 |
| Serous adenocarcinomas | 20 |
| Benign mucinous tumours | 17 |
| Mucinous tumours of borderline malignancy | 5 |
| Mucinous adenocarcinomas | 12 |
| Endometrioid tumours of borderline malignancy | 1 |
| Endometrioid adenocarcinomas | 11 |
| Mesonephroid tumours of borderline malignancy | 1 |
| Mesonephroid adenocarcinomas | 4 |
| Brenner tumours | 7 |
| Mixed epithelial tumours | 4 |
| Undifferentiated adenocarcinomas | 4 |
| Granulosa cell tumours | 5 |
| Androblastomas | 2 |
| Theca cell tumours | 9 |
| Fibromas | 3 |
| Mature teratomas | 6 |
| Metastatic tumours | 5 |
| Total | 240 |

*Reagents*

Rabbit antisera to the beta-subunits of LH and FSH and to PRL, FSH and LH were partly donated by NIAMDD and were also purchased from Calbiochem-Behring Corp. FSH, LH and PRL were purchased from Calbiochem-Behring Corp.

Swine anti-rabbit immunoglobulin antiserum, normal rabbit serum, normal swine serum and rabbit peroxidase-1-antiperoxidase (PAP) were obtained from Dako Immunoglobulins Ltd. whilst diaminobenzidine tetrahydrochloride was purchased from Aldrich Chemical Co.

*Methods*

Serial 5 $\mu$m frozen and paraffin-embedded sections were prepared by routine laboratory procedures and assayed immunocytochemically for detection of hormone binding using the double PAP method as previously reported (Al Timimi *et al.*, 1985). In brief, paraffin sections were deparaffinised in xylene and hydrated through graded alcohols. Endogenous peroxidase activity was blocked by immersing the section in a solution of 1% wt/vol hydrogen peroxide in methanol for 1 h: the sections were then washed for 1 h in Tris buffered saline (TBS) at pH 7.6 and subsequently incubated with a 1:5 dilution of normal swine serum for 5 min in order to reduce non-specific background staining. The sections were then incubated with the appropriate hormone (FSH, LH or PRL), at concentrations of 10–20 $\mu$g ml$^{-1}$ in TBS (pH 7.5) with (vol/vol) 1% absolute ethanol, overnight at 4°C in a moist chamber. Following this the sections were washed and fixed for 5 min in 1% paraformaldehyde to stabilise the hormones at their binding sites. In some sections from each case the step of addition of hormones was omitted to demonstrate endogenous *in vivo* hormone binding. The sections were then washed with TBS and incubated sequentially with the following antisera for the times indicated: (i) rabbit anti-FSH (1:300), rabbit anti-LH (1:300) or rabbit anti-PRL (1:200) for 24 h at 4°C: (ii) swine anti-rabbit immunoglobulin (SAR, diluted 1:50, for 15 min: (iii) PAP soluble complex (1:100) for 30 min: (iv) SAR (1:100) for 15 min: (v) PAP (1:100) for 15 min. Unless noted otherwise all incubations were conducted in a moist chamber at room temperature. The sections were washed with TBS (pH 7.6) after exposure to the primary antisera, SAR and PAP.

The bound hormones were visualised by incubating the slides for 5 min with a filtered freshly prepared solution of 50 mg DAB in 100 ml of TBS (pH 7.6) to which was added 0.02 ml of 30 vol hydrogen peroxide per 100 ml of substrate solution. The slides were then counterstained with haematoxylin, dehydrated to xylene and cover-slipped with permount.

*Specificity controls*

*Tissue controls* Normal pituitary glands were used as positive controls for FSH, LH and PRL staining whilst sections from lung, myocardium and skeletal muscle were used as negative tissue controls.

*Method controls* In every staining run one component of the sequential staining reaction was omitted from at least one section. Usually the primary antiserum was replaced by normal rabbit serum but sometimes SAR or PAP was replaced by diluent buffer or DAB by TBS.

*Absorption controls* These were prepared by mixing a purified hormone preparation with the antiserum to this hormone for 24 h at 4°C prior to the inclusion of the antiserum in the IP procedure.

*Quantitation of results*

The degree of binding was scored on the basis of the proportion of positively staining cases from + to + + +, an admittedly arbitrary and subjective grading system but nevertheless one which, in practice, gave reproducible results.

*Statistical analysis*

All the results obtained were subjected to a chi-square test, a probability level of $P<0.05$, being taken to represent statistical significance.

### Results

A positive reaction for hormone binding was seen as a brown granular precipitate after addition of DAB. All the sections of presumably positive pituitary tissue controls for LH, FSH and PRL gave the expected positive result (Figure 1a) whilst a negative reaction for hormone binding was observed in non-target tissues. The omission of any one component from the sequential immunoperoxidase stain resulted in totally negative results as did the use of antisera which had been previously absorbed by their corresponding hormones (Figure 1b). The results obtained in formalin fixed and paraffin embedded tissues were identical to those seen in frozen sections, both in respect of the number of positively staining cells and the strength of the staining reaction.

Endogenous *in vivo* binding of gonadotrophins and prolactin was seen in many sections of both normal and neoplastic ovarian tissue but the staining was generally weak and occurred in the same sites that subsequently showed binding of exogenous gonadotrophins and prolactin under *in vitro* conditions (Figure 2).

The incidences of a positive reaction for binding of FSH, LH and PRL in both normal and neoplastic ovarian tissue are shown in Table II. The relationship between the presence of these binding sites and the menopausal status of the patient, the histological type of tumour, the histological grade of the neoplasm and the presence or absence of metastases are detailed in Tables II–V.

In the normal ovaries FSH binding was seen in the granulosa cells of the pre-antral and antral

(a)

(b)

**Figure 1** (a) A section of normal human pituitary gland showing FSH-dependent staining in the gonadotrope cells (IP × 500). (b) Immunostaining of human pituitary gland cells abolished by testing with anti-FSH serum which had been previously absorbed with hFSH (IP × 500).

(a)

(b)

**Figure 2** (a) A primary follicle in a normal human ovary. A FSH-binding reaction is present in the ovum and in the granulosa cells (IP × 500). (b) The same follicle as shown in **Figure 2a**. Omission of initial FSH incubation markedly diminishes the staining reaction (IP × 500).

**Table II** Relation between the presence of positive binding sites for FSH, LH and PRL and the morphology of ovarian tissues

| | *Number of cases* | | | *Number staining for FSH binding sites* | | | *Number staining for LH binding sites* | | | *Number staining for PRL binding sites* | | |
|---|---|---|---|---|---|---|---|---|---|---|---|---|
| *Histology* | *pre-menopausal* | *post-menopausal* | *Total* | *pre-menopausal* | *post-menopausal* | *Total* (%) | *pre-menopausal* | *post-menopausal* | *Total* (%) | *pre-menopausal* | *post-menopausal* | *Total* (%) |
| Normal ovaries | 89 | 10 | 99 | 30 | 2 | 32 | 40 | 1 | 41 | 36 | 2 | 38 |
| Benign epithelial tumours | 20 | 20 | 40 | 5 | 7 | 30 | 2 | 5 | 17.5 | 4 | 5 | 22.5 |
| Borderline epithelial tumours | 9 | 9 | 18 | 3 | 4 | 39 | 3 | 2 | 28 | 2 | 4 | 33 |
| Malignant epithelial tumours | 15 | 38 | 53 | 6 | 21 | 51 | 5 | 12 | 32 | 7 | 16 | 43 |
| Sex cord stromal tumours | 7 | 12 | 19 | 2 | 5 | 37 | 3 | 5 | 42 | 2 | 4 | 31.5 |
| Benign teratomas | 5 | 1 | 6 | 0 | 0 | 0 | 0 | 0 | 0 | 0 | 0 | 0 |
| Metastatic tumours | 0 | 5 | 5 | — | 0 | 0 | — | 0 | 0 | — | 0 | 0 |

**Table III** Relationship between presence of positive FSH, LH and PRL binding sites and histological type of tumour

| *Tumour type* | *Number of cases* | *FSH–Positive* No. | *FSH–Positive* (%) | *LH–Positive* No. | *LH–Positive* (%) | *PRL–Positive* No. | *PRL–Positive* (%) |
|---|---|---|---|---|---|---|---|
| *Common epithelial tumours:* | | | | | | | |
| Serous | 45 | 19 | 42 | 11 | 24.5 | 14 | 31 |
| Mucinous | 34 | 12 | 35 | 10 | 29.5 | 14 | 41 |
| Endometrioid | 12 | 5 | 41.5 | 3 | 25 | 3 | 25 |
| Mesonephroid | 5 | 4 | 80 | 2 | 40 | 3 | 60 |
| Brenner | 7 | 2 | 28.5 | 1 | 14 | 1 | 14 |
| Mixed epithelial | 4 | 2 | 50 | 1 | 25 | 1 | 25 |
| Undifferentiated | 4 | 2 | 50 | 1 | 25 | 2 | 50 |
| *Sex cord stromal tumours:* | | | | | | | |
| Granulosa cell | 5 | 2 | 40 | 2 | 40 | 2 | 40 |
| Androblastomas | 2 | 2 | 100 | 2 | 100 | 1 | 50 |
| Thecomas | 9 | 3 | 33 | 4 | 44.5 | 3 | 33 |
| Fibromas | 3 | 0 | 0 | 0 | 0 | 0 | 0 |

**Table IV** Relationship between histological grade of malignant epithelial tumours and presence of positive FSH, LH and PRL binding sites

| *Histological grading* | *Number of cases* | *FSH–Positive* No. | *FSH–Positive* (%) | *LH–Positive* No. | *LH–Positive* (%) | *PRL–Positive* No. | *PRL–Positive* (%) |
|---|---|---|---|---|---|---|---|
| Well differentiated | 21 | 11 | 52 | 8 | 38 | 11 | 52 |
| Moderately differentiated | 17 | 7 | 41 | 4 | 23.5 | 7 | 41 |
| Poorly differentiated | 15 | 9 | 60 | 5 | 33 | 5 | 33 |

**Table V** Relationship between presence of positive FSH, LH and PRL binding sites and metastatic status

| *Metastatic status* | *Number of cases* | *FSH–Positive* No. | *FSH–Positive* (%) | *LH–Positive* No. | *LH–Positive* (%) | *PRL–Positive* No. | *PRL–Positive* (%) |
|---|---|---|---|---|---|---|---|
| Metastases present | 30 | 15 | 50 | 10 | 33 | 13 | 43 |
| Metastases absent | 23 | 12 | 52 | 7 | 30 | 10 | 43 |

follicles (Figure 2a) but was minimal or absent in atretic follicles. The binding sites in the reactive granulosa cells appeared to be localised in the cytoplasm or, in a minority of cases, in the nucleus. Binding of FSH to stromal cells was also seen. LH binding was seen in the granulosa cells of large antral follicles and in the stromal cells. Within the granulosa cells the pattern of binding was similar to that of FSH binding though LH binding appeared to be purely cytoplasmic in the stromal cells. LH binding was also seen in both granulosa cells and thecal cells of corpora lutea. PRL binding followed

**Figure 3** A corpus luteum in a normal human ovary. The lutein cells show a uniformly positive staining for PRL-binding (IP × 370).

(a)

(b)

**Figure 4** (a) An endometrioid adenocarcinoma of the ovary showing a positive staining reaction for FSH-binding in epithelial and periepithelial stromal cells (IP × 500). (b) A section of the same tumour as shown in **Figure 4a**: initial FSH incubation was omitted in order to show *in vivo* binding of endogenous FSH (IP × 500).

the general pattern of LH binding but a notably strong reaction was observed in the luteinised cells of corpora lutea (Figure 3).

Of the 111 epithelial ovarian tumours studied, 46 (41.4%) stained positively for FSH binding, 29 (26%) for LH binding and 38 (34%) for PRL binding. As compared with normal ovarian tissue or with benign epithelial tumours there was a statistically significant excess of FSH binding in malignant epithelial tumours whilst the incidence of PRL binding was significantly greater in malignant epithelial ovarian tumours than in benign epithelial neoplasms of the ovary. There was no significant correlation between the presence of hormone binding sites and the menopausal status of the patient, the histological type of epithelial ovarian tumour, the degree of malignancy of ovarian epithelial tumours or with the presence or absence of metastatic disease.

Hormone binding sites were not demonstrated in mature ovarian teratomas or in metastatic tumours of the ovary but in sex cord stromal tumours, FSH binding was demonstrable in 37% of cases, LH binding in 42% and PRL binding in 31.5%.

Within ovarian neoplasms DAB granules were seen principally in the cytoplasm of the neoplastic epithelial cells (Figures 4 and 5) but in some cases staining was restricted to focal areas of the tumour cell membrane whilst in others staining was predominantly nuclear. A striking feature of the pattern with ovarian neoplasms was the marked heterogeneity of the tumour cells for binding sites to all the studied hormones, strongly staining cells being admixed with cells which gave totally negative staining reactions (Figure 6).

### Discussion

In this study FSH, LH and PRL binding sites have been demonstrated by immunohistochemical techniques in a considerable proportion of normal human ovaries and in many ovarian epithelial tumours. Our finding that formalin-fixed, paraffin embedded tissue sections can be used to detect both *in vivo* and *in vitro* binding sites is in accord with the results of other immunohistochemical studies of gonadotrophin binding sites in rat testes (Childs *et al.*, 1978; Rajaniemi *et al.*, 1981*b*) and rat ovaries (Petrusz & Uhlahrik, 1973; Petrusz, 1974; Petrusz & Sar, 1978) and with studies of PRL binding sites in rat ovaries (Nolin, 1978, 1980; Dunaif *et al.*, 1977, 1982) in human, dog and rat prostatic tissue (Eletreby & Mahrous, 1979; Witorsch, 1978, 1979*a*, 1979*b*; Purnell *et al.*, 1982), dog breast tissue

(a)

(b)

**Figure 5** (a) A serous tumour of borderline malignancy stained for PRL-binding (IP × 500). (b) The same tumour as shown in **Figure 5a**: initial PRL incubation was omitted in order to show *in vivo* binding of endogenous PRL (IP × 500).

(a)

**Figure 6** A Brenner tumour stained for FSH-binding. There is a markedly heterogenous pattern of tumour cell staining (IP × 630).

(Eletreby & Mahrous, 1979) and mouse adrenal gland (McDonough & Ewig, 1982). Dhadley and Walker (1983) were, however, unable to detect PRL binding in paraffin-embedded sections of human breast tissue and advocated the sole usage of frozen sections: it appears therefore that the stability of binding sites for PRL varies from organ to organ.

The validity of immunoperoxidase techniques for the demonstration of hormone receptor sites has been subjected to stringent criticism (Zehr *et al.*, 1981; McCarty *et al.*, 1981; Underwood, 1983) but we have elsewhere countered these arguments (Al-Timimi *et al.*, 1985) and have pointed out that immunohistochemical techniques appear to demonstrate hormone binding to specific recognition sites. It could, of course, be argued that the technique used here simply demonstrates sites of non-specific absorption but this would leave open the question as to why such non-specific binding is not seen in non-target tissues. The specific recognition sites shown by immunohistochemical techniques probably do not coincide in their entirety with the receptors measured by biochemical cytosol assays but nevertheless given an equally characteristic picture of the hormone-binding capacity of particular cells and tissues. This is emphasised by a comparison of our findings with those reported by workers using biochemical techniques to demonstrate FSH, LH and PRL receptors for our results are very similar to those obtained by Kammerman (1980), Kammerman *et al.* (1981) and Rajaniemi *et al.* (1981). Our results in respect to PRL binding in normal human ovarian tissue are also in accord with previous biochemical studies (Poindexter *et al.*, 1979; McNeilly *et al.*, 1980): there have been no previous reports of the frequency of PRL binding in ovarian epithelial tumours but our results for neoplasms of this type correspond very closely to reports of the incidence of biochemically assayable PRL receptors in human breast carcinomas (Di Carlo *et al.*, 1980; Turcot-Lemay & Kelly, 1982).

In this study those ovarian epithelial neoplasms showing a positive reaction for FSH, LH or PRL binding showed a striking degree of heterogeneity with strongly staining cells admixed with totally negative cells. This was not unexpected but in addition, however, ovarian adrenocarcinomas were heterogenous in respect to the cellular localisation of bound gonadotrophins and prolactin. A similar heterogeneity of cellular staining pattern has previously been noted in rat prostatic carcinomas (Witorsch, 1979*a, b*), in human prostatic carcinoma (Purnell *et al.*, 1982) and in human breast carcinomas (Paterson *et al.*, 1982; Purnell *et al.*, 1982; Dhadley & Walker, 1983). This variable pattern of staining for gonadotrophin and prolactin binding sites suggests that there may be subpopulations of ovarian carcinoma cells with differing hormone binding abilities or under different hormonal controls. Until recently the dogma has been that the binding sites for gonadotrophins, like those for other protein hormones, are exclusively present in the limiting membranes of target tissue cells. Recent studies have, however, contradicted this traditional belief by showing that not only the plasma membranes but also various intracellular organelles contain gonadotrophin receptors (Rao *et al.*, 1981; Rajandran & Menon, 1983). Immunoperoxidase studies have also shown that intracellular prolactin is present in rat ovaries (Dunaif *et al.*, 1977, 1982; Nolin, 1978, 1980) and in human breast and prostatic tissue (Purnell *et al.*, 1982) whilst intracellular gonadotrophins have been demonstrated in rat ovary (Petrusz & Uhlarik, 1973; Petrusz, 1974; Petrusz & Sar, 1978) and human prostate (Sibley, 1981).

Our findings in respect to the clinical or prognostic value of the demonstration of gonadotrophin and prolactin binding sites were disappointing. It would have been expected that well differentiated epithelial tumours were more likely to have demonstrable hormone binding sites than would poorly differentiated neoplasms but this proved not to be the case. It is worth noting, however, that the strength of the reaction obtained did vary considerably with the degree of tumour differentiation. Thus, the staining reaction for FSH binding was strong (+++) in 54% of well differentiated tumours showing binding for this hormone but attained this degree of staining intensity in only 22% of positively reacting neoplasms which were poorly differentiated. The equivalent figures for LH binding were 37.5 and 0% whilst those for prolactin binding were 36 and 0%. These figures suggest that whilst the number of cells with hormone binding sites does not alter with decreasing differentiation of an ovarian tumour the number of binding sites in each individual cell does, there being a progressive decrease in staining intensity with decreasing degrees of cellular differentiation.

The incidence of FSH binding was significantly higher in malignant epithelial neoplasms than in either normal ovaries or benign epithelial neoplasms. This observation can, of course, be interpreted in several ways but would certainly tend to support the view that FSH plays a role in ovarian tumourigenesis. It has traditionally been thought, however, that if FSH does indeed play a role in this respect it does so by increasing the number of inclusion cysts derived from the ovarian surface epithelium rather than by promoting malignant change in such cysts. The observation that an increased incidence of FSH-binding was noted in malignant but not in benign epithelial neoplasms of the ovary would tend to argue against this concept.

We wish to thank the many gynaecologists who allowed us to study their patients, in particular Professor V.R. Tindall and Drs. P. Donnai and D.W. Warrell. We are also very grateful to the National Institute of Arthritis, Metabolism and Digestive Disease, Bethesda MD, USA for the gift of pituitary hormones and antisera to these hormones.

### References

AL-TIMIMI, A., BUCKLEY, C.H. & FOX, H. (1985). An immunohistochemical study of the incidence and significance of sex steroid hormone binding sites in normal and neoplastic human ovarian tissue. *Intl. J. Gynecol. Pathol.*, **4**, 24.

BEAMER, W.G. (1980). Endocrinology of ovarian tumors. In *Biology of Ovarian Neoplasia*, Murphy, E.D. & Beamer, W.G. (eds) p. 82. UICC Technical Report Series, Geneva, Vol. 50, Report No. 11.

CHILDS, G.V., HON, C., RUSSELL, L.R. *et al.* (1978). Subcellular localization of gonadotropins and testosterone in the developing fetal rat testis. *J. Histochem. Cytochem.*, **26**, 545.

DAVY, M., TORJESEN, P.A. & AAKVANG, A. (1977). Demonstration of an FSH receptor in a functioning granulosa cell tumour. *Acta. Endocrinol.*, **86**, 615.

DHADLEY, M.S. & WALKER, R.A. (1983). The localisation of prolactin binding sites in human breast tissue. *Int. J. Cancer*, **31**, 433.

DI CARLO, R., MUCCIOLI, G., CONTI, G., REBOANI, C. & DI CARLO, F. (1980). Estrogen and prolactin receptor concentrations in human breast tumours. In *Pharmacological Modulation of Steroid Action*, Genazzani, E. *et al.* (eds) p. 261 Raven Press: New York.

DUNAIF, A.E., ZIMMERMAN, E.A., FRANKZ, A.G. *et al.* (1977). Prolactin and its receptor. Intracellular localization in the ovary by immunoperoxidase technique. *Clin Res.* **25**, 293A.

DUNAIF, A.E., ZIMMERMAN, E.A., FRIESEN, H.G. & FRANTZ, A.G. (1982). Localization of prolactin receptor and prolactin in the rat ovary by immunocytochemistry. *Endocrinology*, **110**, 1465.

ELETREBY, M.F. & MAHROUS, A.T. (1979). Immunocytochemical technique for detection of prolactin and growth hormone in hyperplastic and neoplastic lesions of dog prostrate and mammary gland. *Histochemistry*, **64**, 279.

KAMMERMAN, S. (1980). Gonadotropin receptors in normal and neoplastic ovarian tissue. In *Biology of Ovarian Neoplasia,* Murphy, E.D. & Beamer, W.G. (eds) p. 98. UICC Technical Report Series: Geneva, Vol. 50, Report No. 11.

KAMMERMAN, S., DEMOPOULOS, R.I., RAPHAEL, C. & ROSS, J. (1981). Gonadotropic hormone binding to human ovarian tumors. *Hum. Pathol.,* **12,** 886.

McCARTY, K.S. Jr., REINTGEN, D.S., SEIGLER, H.F. & McCARTY, K.S. Sr. (1981). Cytochemistry of sex steroid receptors: a critique. *Breast Cancer Res. Treat.,* **1,** 315.

McDONOUGH, L. B. & EWIG, J.E. (1982). Immunocytochemical localization of prolactin binding sites in mouse adrenal gland. *Comp. Biochem. Physiol.,* **72**A, 259.

McNEILLY, A.S., KERIN, J., SWANSTON, I.A., BRAMLEY, T.A. & BAIRD, D.T. (1980). Changes in the binding of human chorionic gonadotropin/luteinizing hormone, follicle stimulating hormone and prolactin to human corpora lutea during menstrual cycle and pregnancy. *J. Endocrinol.,* **87,** 315.

NOLIN, J.M. (1978). Intracellular prolactin in rat corpus luteum and adrenal cortex. *Endocrinology,* **102,** 402.

NOLIN, J.M. (1980). Incorporation of endogenous prolactin by granulosa cells and dictyate oocytes in the postpartum rat: effect of oestrogen. *Biol. Reprod.,* **22,** 417.

PATERSON, J.A., SALIH, H. & SHIU, R.P.C. (1982). Immunocytochemical and autoradiographic demonstration of prolactin binding to human breast cancer cells in tissue culture. *J. Histochem. Cytochem.,* **30,** 153.

PETRUSZ, P. (1974). Demonstration of gonadotrophin binding sites in the rat ovary by an immunoglobulin-enzyme bridge method. *Eur. J. Obstet. Gynecol. Reprod. Biol.* (Suppl. 4/1), S3.

PETRUSZ, P. & UHLARIK, A. (1973). Light microscopic localization of binding sites for human chorionic gonadotrophin in luteinized rat ovaries by a peroxidase-labelled antibody method. *J. Histochem. Cytochem.,* **21,** 279.

PETRUSZ, P. & SAR, M. (1978). Light microscopic localization of gonadotropin binding sites in ovarian target cells. In *Cell Membrane Receptors for Drugs and Hormones,* Straub, E.W. & Bolis, L. (eds) p. 557. Raven Press: New York.

POINDEXTER, A.N., BUTTRAM, V.C. Jr., BESCH, P.K. & SMITH, R.G. (1979). Prolactin receptors in the ovary. *Fertil. Steril.,* **31,** 273.

PURNELL, D.M., HILLMAN, E.A., HEATFIELD, B.M. & TRUMP, B.F. (1982). Immunoreactive prolactin in epithelial cells of normal and cancerous human breast and prostate detected by the unlabelled antibody peroxidase-antiperoxidase method. *Cancer Res.,* **42,** 2317.

RAJANDRAN, K.G. & MENON, K.M.T. (1983). Evidence for the existence of gonadotropin receptors in the nuclei isolated from rat ovary. *Biochem. Biophys. Res. Commun.,* **111,** 127.

RAJANIEMI, H., KAUPPILA, A., RÖNNBERG, K., SELANDER, K. & PYSTYNEN, P. (1981a). LH (hCG) receptor in benign and malignant tumours of human ovary. *Acta Obstet. Gynecol. Scand.,* Suppl **101,** 83.

RAJANIEMI, H., KARJALAINEN, M., VEIJOLA, M. *et al.* (1981b). Immunocytochemical localization of receptor human chorionic gonadotropin complexes in rat Leydig cells. *J. Histochem. Cytochem.,* **29,** 813.

RAO, C.H., MITRA, S., SANFILIPPO, J. & CARAN, F.R. Jr. (1981). The presence of gonadotropin binding sites in the intracellular organelles of human ovaries. *Am. J. Obstet. Gynecol.,* **139,** 655.

SIBLEY, P.E.C., HARPER, M.E., JOYCE, B.G., PEELING, W.B. & GRIFFITHS, K. (1981). The immunocytochemical detection of protein hormones in human prostatic tissues. *Prostate,* **2,** 175.

TURCOT-LEMAY, L. & KELLY, P.A. (1982). Prolactin receptors in human breast tumors. *J. Natl. Cancer Inst.,* **68,** 381.

UNDERWOOD, J.C.E. (1983). Oestrogen receptor in human breast cancer: review of histopathological correlation and critique of histochemical methods. *Diagnostic Histopathol.,* **6,** 1.

WITORSCH, R.J. (1978). Immunohistochemical studies of prolactin binding in sex accessory organs of the rat. *J. Histochem. Cytochem.,* **26,** 565.

WITORSCH, R.J. (1979*a*). The application of immunoperoxidase methodology for the visualization of prolactin binding sites in human prostate tissue. *Human Pathol.,* **10,** 521.

WITORSCH, R.J. (1979*b*). Immunohistochemical localization of prolactin-binding sites in R3327 rat prostatic cancer cells. *Hormone Res.,* **10,** 268.

ZEHR, D.R., SATAYASWAROOP, P.G. & SHEEHAN, D.M. (1981). Non-specific staining in the immunolocalization of oestrogen receptors. *J. Steroid Biochem.,* **14,** 613.